# Urgent versus scheduled resection in high-grade glioma: patient selection and healthcare utilization in a single-center cohort

**DOI:** 10.1007/s11060-026-05841-8

**Published:** 2026-10-22

**Authors:** Danny A. Mahmoudi, Trent Kite, Eric Singh, Abigail McKenna, Sami Shaikh, Praveer S. Vyas, John Herbst, Rodney E. Wegner, Stephen Karlovits, Alexander Yu, Alexander C. Whiting, Matthew J. Shepard

**Affiliations:** 1 Drexel University College of Medicine, Philadelphia, PA USA; 2 Department of Neurosurgery, Allegheny Health Network Neuroscience Institute, 320 E North Ave, Pittsburgh, PA 15212 USA; 3 Department of Neuro-oncology, Allegheny Health Network Cancer Institute, Pittsburgh, PA USA; 4 Department of Radiation Oncology, Allegheny Health Network Cancer Institute, Pittsburgh, PA USA

**Keywords:** High-grade glioma, Patient selection, Days alive and at home, Healthcare utilization, Surgical timing, Operative pathway

## Abstract

**Purpose:**

Patients with high-grade glioma reach surgery during the initial, index hospitalization (urgent), or through scheduled/outpatient pathways. There is a paucity of literature examining what patients require expedited versus elective surgery. This single-center retrospective study describes complications, utilization, and survival by pathway.

**Methods:**

Adults with WHO grade 3–4 gliomas undergoing primary resection (2021–2026) were classified as urgent (admitted without a planned resection date) or scheduled (discharged and readmitted for planned surgery, or managed exclusively as an outpatient). Primary outcomes were 30-day complications and days alive at home within 30 days of admission for resection (DAH-30). Secondary outcomes were progression free and overall survival. Survival was analyzed with propensity-score overlap weighting and Cox regression.

**Results:**

Of 166 patients, 122 underwent urgent resection and 44 scheduled resections; 40 of 44 (91%) proceeded as planned. Urgent patients more often had midline shift (62% vs. 32%), less often seizure (15% vs. 39%), and lower Karnofsky scores (70 vs. 80); all *p* ≤ 0.002. They had longer index hospitalizations (median 8 vs. 3 days, *p* < 0.001) and fewer days at home (median 15 vs. 27; adjusted difference − 8.9 days, 95% CI − 12.9 to − 4.8). Complication rates (36% vs. 30%; adjusted OR 1.15, 95% CI 0.52–2.62) and overall survival (median 15.4 vs. 19.0 months, *p* = 0.758) did not differ detectably.

**Conclusions:**

Scheduled resection identified a distinct population, recognizable at initial evaluation by preserved performance status and the absence of midline shift. These patients spent more days at home and required shorter hospitalizations; no harm signal was observed. These data suggest that a substantial proportion of patients with newly diagnosed presumed high grade gliomas can be managed effectively in an expedited, outpatient basis.

**Supplementary Information:**

The online version contains supplementary material available at https://doi.org/10.1007/s11060-026-05841-8.

## Introduction

High-grade gliomas (WHO grades 3 and 4) [1] are the most common primary malignant brain tumors in adults, with a challenging prognosis despite multimodal therapy [2, 3]. The current standard of care involves maximal safe surgical resection followed by concurrent and adjuvant chemoradiation, and tumor-treating fields for eligible patients [4–6]. In practice, patients follow two main surgical pathways: urgent resection at initial presentation or discharge with later admission for scheduled surgery [7]. The National Comprehensive Cancer Network guidelines recommend resection “when feasible” but they do not address timing, pathway, or validated selection criteria [8]. Criteria for scheduling surgery remain poorly defined and conflicting reports exist, and a need for further characterization of these pathways has been called for [9–11].

The interval between diagnosis and surgery may affect optimization of care, including use of 5-aminolevulinic acid and awake craniotomy, both of which require advanced planning [7, 12, 13]. Several studies have shown that moderate delays between diagnosis and surgery do not compromise survival in HGG [9, 11, 14], suggesting that the care pathway decision may be more clinically relevant than the time interval itself.

The care pathway is a modifiable institutional protocol and Chan et al. demonstrated substantially lower total charges for elective supratentorial resections [15]. Nair et al. identified emergent surgery as an independent predictor of extended length of stay [16]. Prior work has established feasibility of scheduled resection but has not fully characterized the populations selected by each pathway [7, 15, 17].

If the patients who are routed to scheduled surgery represent a distinct, presentation-identifiable phenotype, then pathway assignment may be informed by clinical features available at initial evaluation. This study described the clinical profiles, perioperative outcomes, utilization, and survival of patients assigned to urgent versus scheduled pathways for newly diagnosed HGG at a single center, with the goal of characterizing who is selected for each pathway and whether that selection carries a detectable cost in complications or survival.

## Methods

### Study design and participants

This single-center retrospective cohort study included adults (≥ 18 years) with newly diagnosed WHO grade 3–4 glioma who underwent primary resection at Allegheny General Hospital (Allegheny Health Network) between January 1, 2021, and July 1, 2026. Patients were excluded if they had undergone prior craniotomy for tumor, were without resection, or had incomplete operative records (Supplementary Fig. S1). Institutional review board approval (2026-060) was obtained with a waiver of informed consent. All comparisons are reported as urgent versus scheduled.

### Exposure definition

The primary exposure was the operative care pathway, determined by the treating team’s management plan at diagnosis, which was based on imaging and clinical presentation. Patients were classified as urgent if they underwent resection during their initial hospital admission or returned for an unplanned resection after discharge without yet establishing with the neurosurgical-oncology service. Patients discharged home and later readmitted for a scheduled resection were classified as scheduled. Scheduled patients who required unplanned surgery before their intended operative date were analyzed in their assigned group.

### Data collection

Baseline variables were abstracted manually from the electronic medical record and included age, sex, 5-item modified frailty index (mFI-5) [18], American Society of Anesthesiologists physical status [19], preoperative Karnofsky Performance Status (KPS) [20], and presenting symptoms. Preoperative MRI variables included eloquent-cortex involvement, maximal tumor diameter, midline shift, and multifocality. Neuronavigation was used in all resections. WHO grade, IDH mutation status, and MGMT promoter methylation were obtained from the postoperative pathology report. Tumors were classified according to the 2021 WHO classification; glioblastoma was defined as IDH-wildtype grade 4. Extent of resection was determined from early postoperative (≤ 72-hour) MRI: gross-total resection was defined as no residual contrast-enhancing tumor; all others were classified as subtotal. Days alive and at home (DAH-30) was adapted from the DAOH framework [21–23] and calculated as 30 minus the sum of inpatient days and post-acute institutional facility days, with the 30-day window beginning at hospital admission rather than at surgery. This anchoring was chosen to capture preoperative inpatient days accrued by urgent patients. Index hospitalization and any readmission within 30 days were included; patients who died within 30 days were assigned a DAH-30 of zero.

Diagnosis was defined as the date of the first imaging study demonstrating the index lesion. The diagnosis date was used only to measure time to surgery, including for the diagnosis-anchored survival analysis.

Corticosteroid exposure was abstracted as the highest dose given at the diagnostic encounter, either a single bolus or a standing daily regimen, in dexamethasone-equivalent mg/day, excluding perioperative and peri-biopsy doses and those for unrelated indications. For scheduled patients, discharge home on corticosteroids before surgery was also recorded.

### Outcomes

Primary endpoints were any perioperative complication within 30 days and DAH-30. Complications were classified as surgical (intracranial hemorrhage, CSF leak, surgical-site infection, new or worsened neurologic deficit, postoperative seizure) or medical (venous thromboembolism, medical infection, or other non-infectious events). New or worsened neurologic deficits were counted; transient deficits were reported separately. Additional utilization measures included index length of stay, discharge disposition, and 30-day readmission. Secondary outcomes were overall survival (OS), defined as time from surgery to death from any cause, and progression-free survival (PFS), defined as time from surgery to radiographic progression per RANO criteria or death. Patients without an event were censored at last follow-up. Pseudoprogression within 12 weeks of radiotherapy was adjudicated by the treating neuro-oncology team per institutional practice [24].

### Statistical analysis

Continuous variables were summarized as median [range] and compared using the Mann–Whitney U test; categorical variables were summarized as n (%) and compared using Fisher’s exact test. Binary outcomes were modeled with logistic regression, Firth penalized for complications. Index length of stay was analyzed with negative binomial regression, and DAH-30 with median regression and bootstrap confidence intervals. Overall and progression-free survival were estimated by Kaplan–Meier and compared by log-rank test. Multivariable Cox models adjusted for age, KPS, extent of resection, and MGMT methylation, with baseline hazard stratified by IDH status. Because the proportional-hazards assumption was not met, the between-pathway difference was also summarized as the difference in restricted mean survival time at 24 months. Propensity-score overlap weighting was prespecified. Prespecified sensitivity analyses included per-protocol, WHO grade 4–only, and diagnosis-anchored models (Table S1). Overall survival was also modeled with time anchored at diagnosis and left truncation at surgery, to address immortal time accrued by scheduled patients during the preoperative interval. Analyses were repeated in patients with IDH-wildtype grade 4 glioblastoma (Table S4). Analyses were performed in R version 4.5.3; two-sided *p* < 0.05 was considered statistically significant.

## Results

### Cohort and baseline characteristics

Of 166 patients, 122 (73%) underwent urgent resection and 44 (27%) underwent scheduled resection. Of the 44 scheduled patients, 40 (91%) (95% CI 79–96%) proceeded as planned; the remaining 4 required earlier unplanned surgery during the waiting interval and are retained in their assigned group.

Urgent patients presented with lower preoperative KPS (70 [10–100] vs. 80 [60–100], *p* < 0.001) and higher prevalence of ASA class IV (17% vs. 4.5%, *p* = 0.042) (Table 1). Age and frailty band did not differ. Pre-resection biopsy was less common in urgent patients (7.4% vs. 39%, *p* < 0.001). Altered mentation (52% vs. 30%, *p* = 0.013), gait disturbance (38% vs. 9.1%, *p* < 0.001), visual changes (25% vs. 6.8%, *p* = 0.008), and headache (47% vs. 23%, *p* = 0.007) were more frequent in urgent patients, whereas seizure was less frequent among urgent patients (15% vs. 39%, *p* = 0.002). Asymptomatic or incidental diagnosis was less common among urgent patients (0.8% vs. 14%, *p* = 0.001).

**Table 1:** Baseline demographic, frailty, and presenting characteristics by care pathway

Characteristic	Overall (*N* = 166)	Urgent (*n* = 122)	Scheduled (*n* = 44)	*p* value
**Demographics & baseline**				
Age, yr	65 [23–84]	66 [23–84]	62.5 [29–79]	0.072
Male sex	88 (53)	64 (52)	24 (55)	0.861
Preoperative KPS	80 [10–100]	70 [10–100]	80 [60–100]	< 0.001
**ASA class**				0.032
I-II	21 (13)	12 (9.8)	9 (20)	0.109
III	122 (73)	89 (73)	33 (75)	0.845
IV	23 (14)	21 (17)	2 (4.5)	0.042
Biopsy performed	26 (16)	9 (7.4)	17 (39)	< 0.001
**Frailty**				
**mFI-5 category**				0.388
Robust/pre-frail	117 (70)	84 (69)	33 (75)	0.564
Frail	37 (22)	27 (22)	10 (23)	1.000
Severely frail	12 (7.2)	11 (9.0)	1 (2.3)	0.186
**Corticosteroids at presentation**				
Any corticosteroid	136 (82)	115 (94)	21 (48)	< 0.001
Dexamethasone-equivalent dose, mg/day	16 [0–24]	16 [0–24]	0 [0–20]	< 0.001
≥ 16 mg/day	114 (69)	102 (84)	12 (27)	< 0.001
Discharged on corticosteroids before surgery		—	31 (70)	
**Presenting symptoms**				
Seizure	35 (21)	18 (15)	17 (39)	0.002
Confusion / altered mentation	77 (46)	64 (52)	13 (30)	0.013
Visual change	34 (20)	31 (25)	3 (6.8)	0.008
Gait disturbance	50 (30)	46 (38)	4 (9.1)	< 0.001
Focal motor deficit	64 (39)	50 (41)	14 (32)	0.367
Asymptomatic / incidental	7 (4.2)	1 (0.8)	6 (14)	0.001
Headache	67 (40)	57 (47)	10 (23)	0.007
Nausea / vomiting	26 (16)	23 (19)	3 (6.8)	0.088
Aphasia	31 (19)	23 (19)	8 (18)	1.000

Continuous variables are median [range], compared with the Mann–Whitney U test; categorical variables are no. (%), compared with Fisher’s exact test. Multi-level variables show an overall p on the header row and a per-level p beneath. Percentages < 10 are shown to one decimal. Corticosteroid dose is the highest dose given at the diagnostic encounter (single bolus or standing daily regimen), in dexamethasone-equivalent mg/day; discharge on corticosteroids before surgery applies to scheduled patients only. ASA, American Society of Anesthesiologists; KPS, Karnofsky Performance Status; mFI-5, 5-item modified frailty index

Corticosteroid use at presentation was more frequent among urgent patients (115 of 122 [94%] vs. 21 of 44 [48%], *p* < 0.001), and presentation dose was higher (16 [0–24] vs. 0 [0–20] mg/day dexamethasone equivalent, *p* < 0.001; Table 1). A dose of 16 mg/day or more was recorded in 102 urgent (84%) and 12 scheduled (27%) patients, and 31 of 44 scheduled patients (70%) were discharged home on corticosteroids to await surgery.

### Tumor characteristics

Urgent patients had larger tumors (48 [10–87] vs. 36 [10–69] mm, *p* < 0.001) and more frequent midline shift (62% vs. 32%, *p* < 0.001) (Table 2). Eloquent-cortex involvement, tumor location, WHO grade 4 histology, IDH mutation status, and MGMT promoter methylation did not differ between groups.

**Table 2:** Radiographic, tumor, and molecular characteristics by care pathway

Characteristic	Overall (*N* = 166)	Urgent (*n* = 122)	Scheduled (*n* = 44)	*p* value
**Radiographic / tumor**				
Eloquent location	88 (53)	62 (51)	26 (59)	0.382
Max tumor diameter, mm	44.5 [10–87]	48 [10–87]	36 [10–69]	< 0.001
Midline shift	90 (54)	76 (62)	14 (32)	< 0.001
Multifocal disease	33 (20)	23 (19)	10 (23)	0.660
**Tumor location**				0.939
Multilobar	51 (31)	37 (30)	14 (32)	0.851
Frontal	46 (28)	33 (27)	13 (30)	0.845
Temporal	43 (26)	31 (25)	12 (27)	0.842
Parietal	19 (11)	15 (12)	4 (9.1)	0.783
Occipital	3 (1.8)	3 (2.5)	0 (0)	0.566
Insular	2 (1.2)	1 (0.8)	1 (2.3)	0.461
Cerebellar	1 (0.6)	1 (0.8)	0 (0)	1.000
Other	1 (0.6)	1 (0.8)	0 (0)	1.000
**Pathology & molecular**				
**WHO grade**,** IDH status**,** and histologic diagnosis**				
Grade 4, IDH-wildtype	152 (92)	115 (94)	37 (84)	
Glioblastoma	144 (87)	108 (89)	36 (82)	
Gliosarcoma	3 (1.8)	3 (2.5)	0 (0)	
Other HGG	3 (1.8)	3 (2.5)	0 (0)	
Astrocytoma	2 (1.2)	1 (0.8)	1 (2.3)	
Grade 4, IDH-mutant	3 (1.8)	2 (1.6)	1 (2.3)	
Astrocytoma	3 (1.8)	2 (1.6)	1 (2.3)	
Grade 3, IDH-mutant	8 (4.8)	4 (3.3)	4 (9.1)	
Astrocytoma	5 (3.0)	2 (1.6)	3 (6.8)	
Oligodendroglioma	3 (1.8)	2 (1.6)	1 (2.3)	
Grade 3, IDH-wildtype	3 (1.8)	1 (0.8)	2 (4.5)	
Astrocytoma	2 (1.2)	1 (0.8)	1 (2.3)	
Other HGG	1 (0.6)	0 (0)	1 (2.3)	
WHO grade 4	155 (93)	117 (96)	38 (86)	0.069
IDH-mutant	11 (6.6)	6 (4.9)	5 (11)	0.162
MGMT methylated	71/154 (46)	51/113 (45)	20/41 (49)	0.718

Histologic diagnosis is the label on the pathology report; under the 2021 WHO classification, all IDH-wildtype grade 4 tumors (*n* = 152) are glioblastoma. MGMT was untested in 12 patients; methylation percentages use the tested denominator. IDH, isocitrate dehydrogenase; MGMT, O6-methylguanine-DNA methyltransferase; WHO, World Health Organization

### Surgical management and perioperative complications

Extent of resection did not differ, with gross-total resection at 39% versus 41% (*p* = 0.859). 5-aminolevulinic acid was used in 30% of urgent and 45% of scheduled cases (*p* = 0.095). Awake craniotomy was less common in urgent cases (6.6% vs. 18%, *p* = 0.036) (Table 3). Any complication within 30 days occurred in 57 patients (34%), 44 (36%) urgent versus 13 (30%) scheduled (*p* = 0.465), and did not differ after adjustment (Firth aOR 1.15, 95% CI 0.52–2.62, urgent versus scheduled). Permanent neurologic deficits persisting to three months occurred in 21 patients (13%) (12% vs. 14%, *p* = 0.796); an additional 13 transient deficits resolved by three months. Individual complication events were infrequent and did not differ by pathway: surgical-site infection 4.9% versus 2.3% (*p* = 0.677), intracranial hemorrhage 5.7% versus 2.3% (*p* = 0.683), CSF leak 4.9% versus 0% (*p* = 0.343), new postoperative seizure 4.1% versus 2.3% (*p* = 1.000), and venous thromboembolism 5.7% versus 2.3% (*p* = 0.683). Unplanned reoperation occurred in 9.8% versus 2.3% (*p* = 0.188), all-cause readmission within 30 days in 14% versus 9.1% (*p* = 0.597), and 30-day mortality in 3.3% versus 0% (*p* = 0.574), none reaching significance (Table 3).

**Table 3:** Surgical management, perioperative complications, and outcomes by care pathway

Characteristic	Overall (*N* = 166)	Urgent (*n* = 122)	Scheduled (*n* = 44)	*p* value
**Hospital course**				
Diagnosis-to-surgery, days	7 [1–290]	5 [1–169]	25 [7–290]	< 0.001
Preoperative LOS, days	3 [0–12]	4 [0–12]	0 [0–3]	< 0.001
Index LOS, days	7 [1–39]	8 [1–39]	3 [2–15]	< 0.001
Postoperative LOS, days	3 [1–27]	4 [1–27]	3 [1–12]	0.002
ICU LOS, days	1 [0–16]	1 [0–16]	1 [0–6]	0.261
Days alive & at home, 30 d (DAH-30)	18 [0–29]	15 [0–29]	27 [0–28]	< 0.001
**Surgical**				
Gross-total resection	66 (40)	48 (39)	18 (41)	0.859
Awake craniotomy	16 (9.6)	8 (6.6)	8 (18)	0.036
5-ALA fluorescence	57 (34)	37 (30)	20 (45)	0.095
**Complications (30-day)**				
Any postoperative complication	57 (34)	44 (36)	13 (30)	0.465
Any surgical complication	47 (28)	37 (30)	10 (23)	0.436
Any medical complication	21 (13)	18 (15)	3 (6.8)	0.289
New/worsened neurologic deficit (permanent)	21 (13)	15 (12)	6 (14)	0.796
Surgical-site infection	7 (4.2)	6 (4.9)	1 (2.3)	0.677
Intracranial hemorrhage	8 (4.8)	7 (5.7)	1 (2.3)	0.683
CSF leak	6 (3.6)	6 (4.9)	0 (0)	0.343
New postoperative seizure	6 (3.6)	5 (4.1)	1 (2.3)	1.000
Venous thromboembolism (post-op DVT/PE)	8 (4.8)	7 (5.7)	1 (2.3)	0.683
**Outcomes**				
Return to OR ≤ 30 d (all cause)	13 (7.8)	12 (9.8)	1 (2.3)	0.188
Readmission ≤ 30 d (all cause)	21 (13)	17 (14)	4 (9.1)	0.597
30-day mortality	4 (2.4)	4 (3.3)	0 (0)	0.574
Non-home discharge	77 (46)	65 (53)	12 (27)	0.004
**Adjuvant timing**				
Surgery-to-radiotherapy, days	36 [17–66]	36 [17–66]	39 [23–62]	0.673
Surgery-to-chemotherapy, days	37 [17–145]	36.5 [17–145]	39.5 [23–144]	0.356

Values are median [range] (Mann–Whitney U) or no. (%) (Fisher’s exact). Deficits are permanent (persisting to 3 months; deficits unresolved at death or last follow-up before 3 months were classified as permanent). Adjuvant timing is among patients with a recorded interval (radiotherapy *n* = 143; chemotherapy *n* = 142). DAH-30, days alive and at home within 30 days; LOS, length of stay; OR, operating room

### Healthcare utilization and days alive at home

**Fig. 1:**
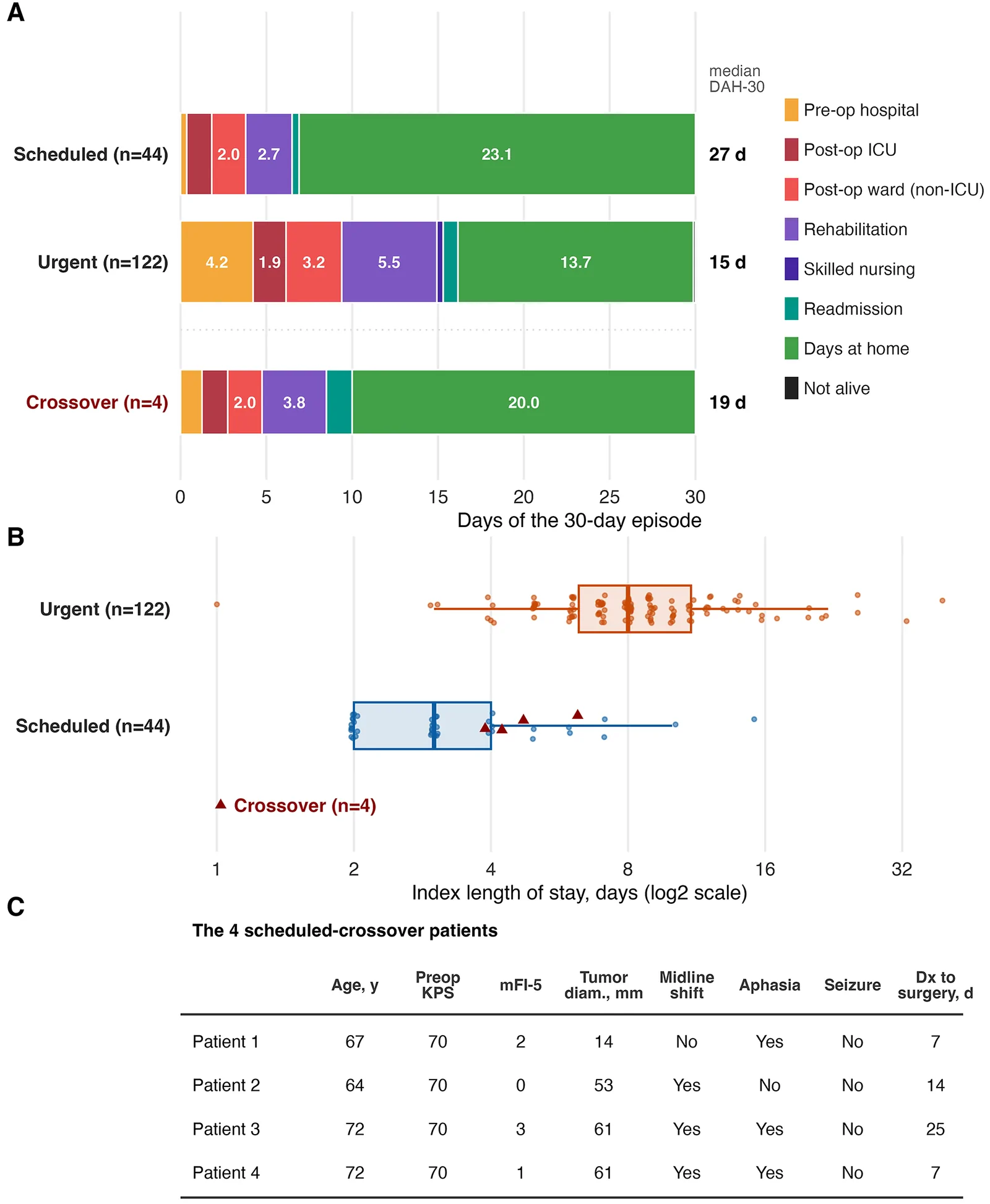
Thirty-day healthcare utilization by care pathway. (**A**) Mean days spent in each location during the first 30 days; each bar totals 30 days, with the median days alive and at home (DAH-30) annotated at right. Median DAH-30 was 15 days for urgent (*n* = 122) and 27 days for scheduled (*n* = 44) patients; a separate bar summarizes the four scheduled-crossover patients. (**B**) Index length of stay by pathway; boxes show the median and interquartile range with individual patients overlaid on a log2 scale, and the four scheduled-crossover patients marked as red triangles. The adjusted index length-of-stay rate ratio was 2.30 (95% CI 1.85–2.86) and the adjusted median DAH-30 difference was − 8.9 days (95% CI − 12.9 to − 4.8), both urgent versus scheduled. The full length-of-stay breakdown is given in Table 3. (**C**) The four scheduled-crossover patients routed to scheduled surgery but requiring earlier unplanned resection by age, preoperative Karnofsky Performance Status (KPS), 5-item modified frailty index (mFI-5), tumor diameter, midline shift, aphasia, seizure at presentation, the diagnosis-to-surgery interval; all four presented with a KPS of 70. DAH-30, days alive and at home; KPS, Karnofsky Performance Status; LOS, length of stay; mFI-5, 5-item modified frailty index

Urgent patients had longer index hospitalizations (median 8 [1–39] vs. 3 [2–15] days, *p* < 0.001; Fig. 1; adjusted and overlap-weighted utilization estimates in Table S2), driven primarily by preoperative days (median 4 vs. 0). In a negative-binomial model, urgent patients had approximately twofold longer index stays (rate ratio 2.30, 95% CI 1.85–2.86, *p* < 0.001); the overlap-weighted difference was 4.6 days (3.4–5.8).

Urgent patients spent fewer days alive at home within 30 days of admission (DAH-30 median 15 [0–29] vs. 27 [0–28], *p* < 0.001; Fig. 1). This difference persisted after adjustment (median difference − 8.9 days, 95% CI − 12.9 to − 4.8) and with overlap weighting (− 7.6 days, − 10.8 to − 4.3).

Non-home discharge was more common among urgent patients (53% vs. 27%, *p* = 0.004; adjusted OR 2.25, 95% CI 1.00–5.06); the overlap-weighted estimate was attenuated (OR 1.71, 0.75–3.90). Despite a shorter diagnosis-to-surgery interval for urgent patients (5 [1–169] vs. 25 [7–290] days, *p* < 0.001), time from surgery to radiotherapy (36 vs. 39 days, *p* = 0.673) and chemotherapy (36.5 vs. 39.5 days, *p* = 0.356) did not differ. Seven patients had a diagnosis-to-surgery interval exceeding 90 days (6 scheduled, 1 urgent). On chart review, all were followed with serial imaging and none was lost to follow-up. Excluding them did not materially change the DAH-30, complication, or survival estimates (− 8.7 days, aOR 1.12, and HR 0.62, respectively).

### Survival

**Fig. 2:**
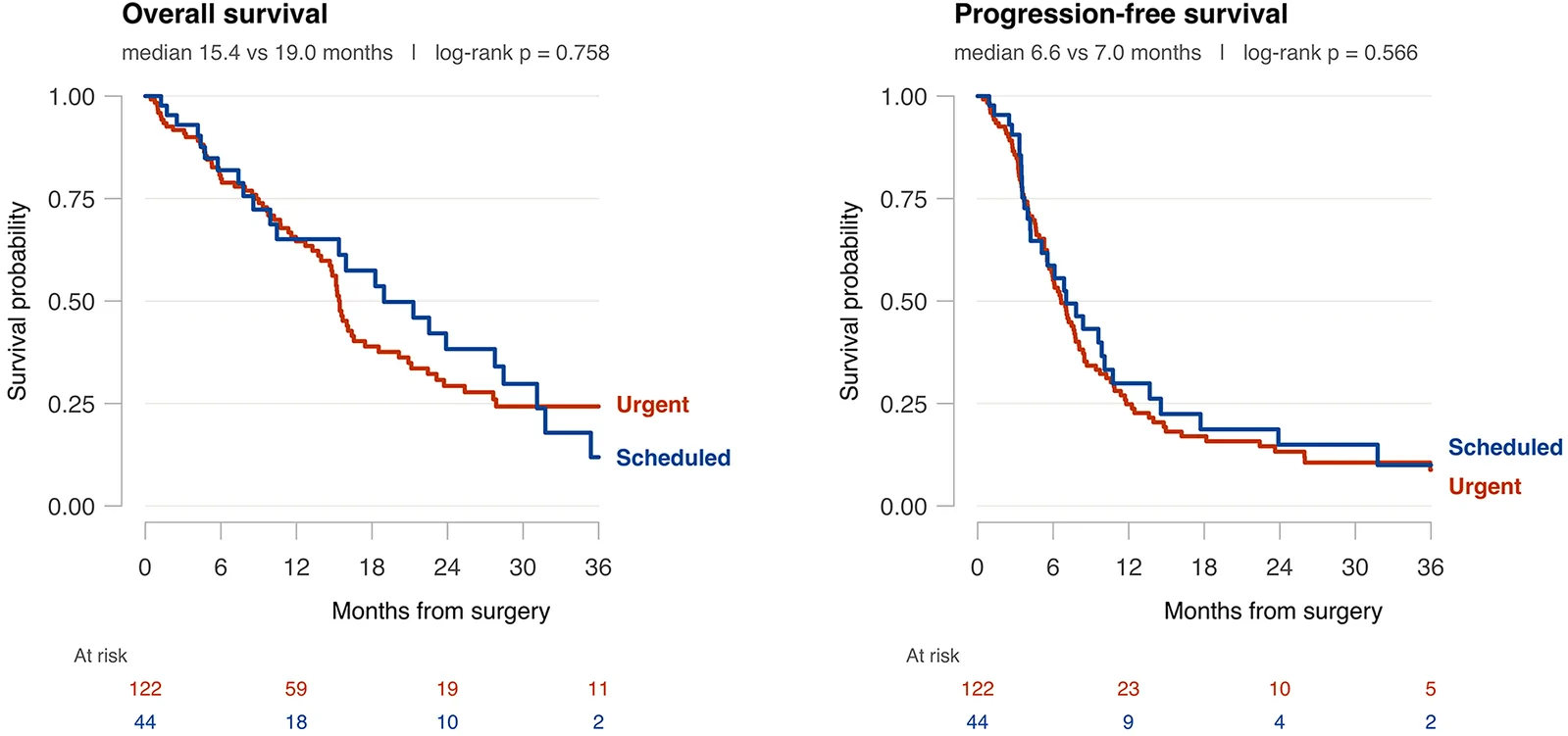
Kaplan–Meier overall (left) and progression-free (right) survival by care pathway. Median overall survival was 15.4 months (urgent) versus 19.0 months (scheduled), log-rank *p* = 0.758; median progression-free survival was 6.6 versus 7.0 months, log-rank *p* = 0.566. Numbers at risk are shown beneath each panel

Median follow-up was 26.2 months by reverse Kaplan–Meier, with 94 deaths. Median overall survival was 15.4 months (95% CI 14.7–18.5) for urgent versus 19.0 months (15.4–31.1) for scheduled patients (log-rank *p* = 0.758; crude HR 1.08, 95% CI 0.68–1.72; Fig. 2). Median progression-free survival was 6.6 months (5.7–8.0) versus 7.0 months (5.1–13.7; *p* = 0.566). In a Cox model adjusted for age, KPS, extent of resection, and MGMT methylation, with baseline hazard stratified by IDH status, the adjusted HR for overall survival was 0.64 (95% CI 0.38–1.10, *p* = 0.105), or 0.67 (95% CI 0.39–1.14) with time anchored at diagnosis and left truncation; for progression-free survival it was 0.77 (0.49–1.24, *p* = 0.285). The shift from the crude estimate reflects adjustment for baseline severity differences between groups. Because the proportional-hazards assumption was not met globally (Table S3), survival was also compared on an absolute scale: the adjusted difference in restricted mean survival time at 24 months was 0.34 months (95% CI − 2.48 to + 3.17).

In the 152 patients with IDH-wildtype grade 4 glioblastoma, urgent patients again spent fewer days at home (adjusted DAH-30 difference − 6.4 days, 95% CI − 10.1 to − 2.7), and complications (aOR 1.05, 95% CI 0.46–2.49) and survival (adjusted HR 0.64, 95% CI 0.37–1.10) did not differ significantly (Table S4).

### Scheduled-crossover subgroup

Four of 44 scheduled patients (9.1%) required surgery before their intended operative date. All four had a preoperative KPS of 70 the median of the urgent arm. Midline shift, aphasia, and tumors exceeding 50 mm in diameter were each present in three of the four (Fig. 1C). None presented with seizure. Three of the four received 16 mg/day dexamethasone equivalent at presentation and were discharged home on corticosteroids before surgery; one received none. Ages ranged from 64 to 72. Although the subgroup is too small for inference (Fig. 1C), these patients shared features characteristic of the urgent phenotype older age, larger tumors, lower performance status, and language deficit suggesting that the clinical markers distinguishing the two pathways may also identify scheduled patients at risk of requiring earlier intervention.

## Discussion

In this cohort, the urgent and scheduled pathways differed substantially in patient characteristics and healthcare utilization but not in perioperative complications, extent of resection, adjuvant timing, or survival. Because pathway assignment is determined by the same clinical features that influence prognosis, any comparison of these groups is inevitably a comparison of two clinical phenotypes. The crude survival difference partly reflected tumor composition: IDH-mutant tumors made up 4.9% of urgent and 11% of scheduled patients, and none of the 11 patients with IDH-mutant tumors died during follow-up. The shift from the crude HR of 1.08 to the adjusted 0.64 is consistent with confounding by indication rather than an independent pathway effect. Neither estimate reached significance, and the adjusted difference in restricted mean survival time at 24 months was 0.34 months (95% CI − 2.48 to + 3.17).

With a median survival of 15.7 months in this cohort, days spent in the hospital are drawn from a limited remaining lifespan; population-level data show that more than a third of glioblastoma patients over 75 spend at least half of their remaining life hospitalized [25]. DAH-30 was chosen as a co-primary endpoint because it captures both survival and time spent outside institutional care within a fixed window [21–23]. Urgent patients had longer index stays, fewer days at home, and more non-home discharges, with no difference in extent of resection. Scheduled patients spent 8.9 more adjusted days at home in the first 30 days (95% CI 4.8–12.9), exceeding the 3-day minimal clinically important difference proposed for this measure [26]. Because DAH-30 was anchored to admission rather than to surgery, part of this difference reflects preoperative inpatient days that urgent patients accrued and scheduled patients did not.

Complication rates did not differ in unadjusted analysis (36% vs. 30%, *p* = 0.465), after Firth penalization (aOR 1.15, 0.52–2.62), or with overlap weighting (OR 1.03, 0.45–2.36). Deng et al. reported higher 30-day mortality and complications after emergency glioma resection, though their comparison focused on truly emergent cases that may not be directly comparable to the pathways defined here [27].

These utilization differences are consistent with prior work on service configuration. Chan et al. [15] found that non-elective supratentorial resections incurred substantially higher charges and lower profit margins. Guilfoyle et al. [17] showed that adopting a planned elective model nearly halved median hospital stay and reduced inpatient costs. In this study, 91% of scheduled patients completed their planned operation; survival, extent of resection, and complication rates did not differ detectably between pathways.

Scheduled patients more frequently underwent awake craniotomy, while 5-ALA use and extent of resection did not differ. Awake craniotomy requires preoperative neuropsychological screening and specialized anesthesia planning incompatible with urgent timelines. By contrast, 5-ALA requires only oral administration 2–4 h preoperatively, a threshold achievable even in urgent settings [12]. Moniz-Garcia et al. demonstrated that a standardized awake craniotomy program predicated on elective scheduling reduced mean length of stay by nearly a day, with mean savings of $7,089 per patient; extent of resection and complication rates were unchanged [13]. Sun et al. found that transitioning from emergency to elective surgery enhanced adjunct use and extent of resection [7]; our data support this access advantage for planning-intensive techniques while confirming that low-barrier adjuncts can be deployed in either pathway. Surgery-to-radiotherapy (36 vs. 39 days, *p* = 0.673) and surgery-to-chemotherapy (36.5 vs. 39.5 days, *p* = 0.356) intervals did not differ and fell within the 3–6-week window recommended by American Society for Radiation Oncology ASTRO [28]. Press et al. [29] and Buszek et al. [30] identified worse survival with radiotherapy started before 3 weeks or, after gross-total resection, beyond 8 weeks. Both pathways in this study delivered adjuvant therapy within these intervals.

Seizure was more frequent among scheduled patients (39% vs. 15%, *p* = 0.002). At diagnosis, seizure has been described as an independent favorable prognostic marker in glioblastoma, with a pooled hazard ratio of 0.71 (95% CI 0.63–0.81) in meta-analysis [31], and Dimova et al. found tumor-associated epilepsy independently associated with improved survival and a distinct inflammatory signature [32]. In this cohort, including seizure as a covariate did not materially alter the pathway term, and seizure was not independently prognostic (HR 1.18, 95% CI 0.65–2.15).

Corticosteroid exposure also differed by pathway and tracked presentation severity. Near-universal use among urgent patients (94%, with 84% at 16 mg/day or more) is consistent with the greater mass effect that defined that phenotype. Use was less frequent and at lower doses among scheduled patients.

The scheduled pathway comprised patients with preserved performance status, smaller tumors, absence of midline shift, and more frequent seizure presentation a profile identifiable through initial clinical and imaging assessment. Of these, 91% (40/44) proceeded as planned, and scheduled patients spent 8.9 more adjusted days at home in the first 30 days. In a disease measured in months, this difference is proportionally substantial: it represents time during which patients are home, functional, and able to consult with family, consider treatment options, and prepare for the demands of multimodal therapy. A high-grade glioma diagnosis confronts patients with decisions that carry lasting consequences extent of resection, enrollment in clinical trials, goals of care. The ability to make those decisions from home, with adequate time and support, is a material component of individualized surgical care. The scheduled pathway, by its nature, affords this interval; urgent presentation forecloses it. Complications, extent of resection, adjuvant timing, and survival did not differ detectably between groups. These observations reflect institutional selection practices rather than formalized criteria. Whether patients currently managed urgently could be safely scheduled requires prospective validation, but the clinical features that defined the scheduled phenotype in this cohort preserved performance, no midline shift, smaller tumor burden offer a starting point for that evaluation.

It should be noted that the patients included in this cohort were treated by a variety of surgeons and not exclusive to neurosurgical oncologists. These data therefore suggest that the substantial majority of patients who are deemed stable and fit for expedited, outpatient resection would be able to benefit from consultation with a dedicated tumor surgeon. While only 44/166 (27%) of patients were scheduled for elective surgery, it is possible that a subset of the urgent cases herein would have been able to be effectively managed on an outpatient basis. 15.6% of patients in the urgent cohort had a KPS ≥ 80, tumors <5 cm without midline shift. Patients with these characteristics are more likely to benefit from expedited outpatient surgery. In order to effectively manage these patients as an outpatient, multidisciplinary clinics comprised of nurse navigators, neuro-oncologists, radiation oncologists and neurosurgeons are tantamount to delivering enhanced, expedited care and should be supported by healthcare systems.

### Limitations

Confounding by indication is inherent to this design: pathway assignment is driven by clinical judgment, and sicker patients are more likely to undergo urgent surgery. Propensity-score overlap weighting and multivariable adjustment addressed measured confounders, but residual confounding by unmeasured variables cannot be excluded, and would be expected to bias utilization comparisons in favor of the scheduled arm. Molecular characterization was limited to routinely available markers; TERT promoter status and CDKN2A/B alterations were not assessed. Because diagnosis was dated to the first imaging study demonstrating the lesion, the diagnosis-to-surgery interval in seven patients included a period of surveillance or observation before resection. The crossover subgroup (*n* = 4) permits only descriptive reporting. The single-center setting limits generalizability but ensured uniform surgical technique, adjunct access, and data collection.

### Conclusion

Scheduled resection at this center identified a distinct, presentation-recognizable phenotype that spent more days at home and required shorter hospitalizations; complications, extent of resection, adjuvant timing, and survival did not differ detectably. The actionable question these data motivate is which patients currently managed through the urgent pathway share this profile closely enough to be scheduled a question that requires prospective, multi-institutional evaluation.

## Supplementary information

Below is the link to the electronic supplementary material.

Supplementary Material 1

## Data Availability

Owing to institutional and patient-privacy regulations, the data are not publicly available; de-identified data may be shared by the corresponding author on reasonable request and with institutional approval.

## Code availability

The analysis code that generates every table, figure and supplementary item reported here was drafted and debugged with the assistance of Claude (Anthropic), run in R 4.5.3, and verified by the authors. It is available from the corresponding author on reasonable request.

## Declarations

### Reporting guideline

This study is reported in accordance with the STROBE statement for observational research.
