# Supplementary material for: Urgent versus scheduled resection in high-grade glioma: patient selection and healthcare utilization in a single-center cohort
Source: J Neurooncol. 2026 Oct 22;180(2):29. doi: 10.1007/s11060-026-05841-8 (PMC13690921; doi:10.1007/s11060-026-05841-8)
Supplement: Supplementary file 1 [file 11060_2026_5841_MOESM1_ESM.docx]

## 1.1. Figure S1. Participant flow

Screening, exclusions, and pathway assignment. Analytic cohort n = 166 (urgent 122, scheduled 44). The scheduled arm comprises 40 patients who underwent surgery as planned and 4 who required earlier unplanned resection.

## 1.2. Table S1. Pathway effect on overall survival: primary and prespecified sensitivity analyses

| **Analysis** | **Patients / deaths** | **Scale** | **Estimate (95% CI)** | **p** |
| --- | --- | --- | --- | --- |
| Kaplan–Meier, log-rank (primary) | 166 / 94 | Median OS, months | 15.4 vs 19.0 | 0.758 |
| Crude Cox model | 166 / 94 | HR | 1.08 (0.68–1.72) | 0.758 |
| Adjusted Cox model | 154 / 88 | HR | 0.64 (0.38–1.10) | 0.105 |
| Per-protocol (crossovers reclassified as urgent) | 154 / 88 | HR | 0.77 (0.43–1.38) | 0.384 |
| WHO grade 4 only | 144 / 86 | HR | 0.64 (0.37–1.10) | 0.107 |
| Diagnosis-anchored (left-truncated) | 154 / 88 | HR | 0.67 (0.39–1.14) | 0.137 |
| Adjusted RMST difference at 24 months | 154 / 88 | months | +0.34 (−2.48 to +3.17) | 0.813 |

HR, hazard ratio (urgent versus scheduled); values below 1 indicate lower hazard in the urgent group. Adjusted models include age, KPS, extent of resection, and MGMT methylation, with baseline hazard stratified by IDH status; no IDH-mutant patient died, so an IDH hazard ratio was not estimable. Adjusted analyses are complete-case (MGMT untested in 12 patients). The per-protocol analysis reclassified the four scheduled patients who required earlier surgery as urgent. The diagnosis-anchored model measured survival from diagnosis with left truncation at surgery. RMST, restricted mean survival time to 24 months (urgent minus scheduled, months), estimated by regression on jackknife pseudo-observations with robust (HC3) standard errors, adjusted for the same covariates. p values are not adjusted for multiplicity. KPS, Karnofsky Performance Status; OS, overall survival.

## 1.3. Table S2. Adjusted and overlap-weighted estimates for complications and healthcare utilization

| **Outcome** | **Analysis** | **Scale** | **Estimate (95% CI)** |
| --- | --- | --- | --- |
| Index length of stay | Adjusted | rate ratio | 2.30 (1.85–2.86) |
| Index length of stay | Overlap-weighted | difference in means, days | 4.6 (3.4–5.8) |
| DAH-30 | Adjusted | difference in medians, days | −8.9 (−12.9 to −4.8) |
| DAH-30 | Overlap-weighted | difference in means, days | −7.6 (−10.8 to −4.3) |
| Non-home discharge | Adjusted | odds ratio | 2.25 (1.00–5.06) |
| Non-home discharge | Overlap-weighted | odds ratio | 1.71 (0.75–3.90) |
| Any 30-day complication | Adjusted | odds ratio (Firth) | 1.15 (0.52–2.62) |
| Any 30-day complication | Overlap-weighted | odds ratio | 1.03 (0.45–2.36) |

All estimates compare urgent with scheduled patients; for these adverse outcomes, ratios above 1, longer stays, and fewer days at home indicate less favorable values in the urgent group. Adjusted models: any 30-day complication, Firth-penalized logistic regression (age, KPS, tumor diameter, mFI-5, eloquent location); non-home discharge, logistic regression (age, KPS, mFI-5); index LOS, negative binomial regression (age, KPS, tumor diameter, mFI-5); DAH-30, median regression with bootstrap standard errors (2,000 replicates; age, KPS, tumor diameter, mFI-5, ASA IV). Overlap-weighted estimates use propensity-score overlap weights from a logistic model including age, KPS, tumor diameter, mFI-5, midline shift, and ASA IV. The adjusted and overlap-weighted DAH-30 rows estimate a difference in medians and in means, respectively. ASA, American Society of Anesthesiologists; DAH-30, days alive and at home within 30 days; KPS, Karnofsky Performance Status; LOS, length of stay; mFI-5, 5-item modified frailty index.

## 1.4. Table S3. Proportional-hazards diagnostics for the adjusted Cox models

| **Model** | **Term** | **χ²** | **df** | **p** |
| --- | --- | --- | --- | --- |
| Overall survival | Pathway | 2.613 | 1 | 0.106 |
|  | Age | 1.643 | 1 | 0.200 |
|  | KPS | 13.491 | 1 | <0.001 |
|  | Extent of resection | 1.357 | 1 | 0.244 |
|  | MGMT | 0.655 | 1 | 0.418 |
|  | Global test | 14.517 | 5 | 0.013 |
| Progression-free survival | Pathway | 0.242 | 1 | 0.623 |
|  | Age | 0.124 | 1 | 0.725 |
|  | KPS | 9.946 | 1 | 0.002 |
|  | Extent of resection | 6.764 | 1 | 0.009 |
|  | MGMT | 0.669 | 1 | 0.413 |
|  | Global test | 14.213 | 5 | 0.014 |

Scaled Schoenfeld residual tests (n = 154, complete case). The pathway term did not violate proportional hazards in either model; the global test was significant, driven by KPS (and extent of resection for progression-free survival). The restricted mean survival time difference (Table S1) is therefore reported as an absolute measure that does not assume proportional hazards. KPS, Karnofsky Performance Status.

## 1.5. Table S4. Adjusted and overlap-weighted estimates in patients with IDH-wildtype grade 4 glioblastoma

| **Outcome** | **Analysis** | **Scale** | **Estimate (95% CI)** |
| --- | --- | --- | --- |
| Index length of stay | Adjusted | rate ratio | 2.31 (1.84–2.91) |
| Index length of stay | Overlap-weighted | difference in means, days | 4.8 (3.6–6.0) |
| DAH-30 | Adjusted | difference in medians, days | −6.4 (−10.1 to −2.7) |
| DAH-30 | Overlap-weighted | difference in means, days | −7.0 (−10.4 to −3.6) |
| Non-home discharge | Adjusted | odds ratio | 2.06 (0.87–4.85) |
| Non-home discharge | Overlap-weighted | odds ratio | 1.48 (0.62–3.51) |
| Any 30-day complication | Adjusted | odds ratio (Firth) | 1.05 (0.46–2.49) |
| Any 30-day complication | Overlap-weighted | odds ratio | 0.94 (0.39–2.29) |
| Overall survival | Kaplan–Meier | median, months | 15.2 vs 15.9 |
| Overall survival | Crude Cox model | hazard ratio | 0.85 (0.53–1.36) |
| Overall survival | Adjusted Cox model | hazard ratio | 0.64 (0.37–1.10) |
| Overall survival | Diagnosis-anchored (left-truncated) | hazard ratio | 0.65 (0.38–1.12) |
| Overall survival | Adjusted RMST difference at 24 months | months | +0.65 (−2.44 to +3.74) |

All estimates compare urgent with scheduled patients among the 152 patients with IDH-wildtype grade 4 glioblastoma (115 urgent, 37 scheduled; 92 deaths). Models and covariates are those of Tables S1 and S2, without IDH stratification; adjusted Cox models are complete-case (n = 141, 86 deaths). For adverse outcomes, ratios above 1, longer stays, and fewer days at home indicate less favorable values in the urgent group; hazard ratios below 1 indicate lower hazard in the urgent group. DAH-30, days alive and at home within 30 days; RMST, restricted mean survival time.
